# Targeting melanoma growth and viability reveals dualistic functionality of the phosphonothionate analogue of carba cyclic phosphatidic acid

**DOI:** 10.1186/1476-4598-9-140

**Published:** 2010-06-09

**Authors:** Molly K Altman, Vashisht Gopal, Wei Jia, Shuangxing Yu, Hassan Hall, Gordon B Mills, A Cary McGinnis, Michael G Bartlett, Guowei Jiang, Damian Madan, Glenn D Prestwich, Yong Xu, Michael A Davies, Mandi M Murph

**Affiliations:** 1 Department of Pharmaceutical and Biomedical Sciences, The University of Georgia, College of Pharmacy, 250 W. Green Street, Athens, Georgia 30602, USA; 2 Department of Melanoma Medical Oncology, The University of Texas MD Anderson Cancer Center, 7455 Fannin, 1 SCRB 2.3019, Houston, TX 77054, USA; 3 Department of Systems Biology, The University of Texas MD Anderson Cancer Center, 1515 Holcombe Blvd., Houston, TX 77030, USA; 4 Department of Medicinal Chemistry, The University of Utah, 419 Wakara Way, Suite 205, Salt Lake City, UT 84108, USA; 5 Echelon Biosciences Inc., 675 Arapeen Dr., Suite 302, Salt Lake City, UT 84108, USA

## Abstract

**Background:**

Although the incidence of melanoma in the U.S. is rising faster than any other cancer, the FDA-approved chemotherapies lack efficacy for advanced disease, which results in poor overall survival. Lysophosphatidic acid (LPA), autotaxin (ATX), the enzyme that produces LPA, and the LPA receptors represent an emerging group of therapeutic targets in cancer, although it is not known which of these is most effective.

**Results:**

Herein we demonstrate that thio-ccPA 18:1, a stabilized phosphonothionate analogue of carba cyclic phosphatidic acid, ATX inhibitor and LPA1/3 receptor antagonist, induced a marked reduction in the viability of B16F10 metastatic melanoma cells compared with PBS-treated control by 80-100%. Exogenous LPA 18:1 or D-sn-1-O-oleoyl-2-O-methylglyceryl-3-phosphothioate did not reverse the effect of thio-ccPA 18:1. The reduction in viability mediated by thio-ccPA 18:1 was also observed in A375 and MeWo melanoma cell lines, suggesting that the effects are generalizable. Interestingly, siRNA to LPA3 (siLPA3) but not other LPA receptors recapitulated the effects of thio-ccPA 18:1 on viability, suggesting that inhibition of the LPA3 receptor is an important dualistic function of the compound. In addition, siLPA3 reduced proliferation, plasma membrane integrity and altered morphology of A375 cells. Another experimental compound designed to antagonize the LPA1/3 receptors significantly reduced viability in MeWo cells, which predominantly express the LPA3 receptor.

**Conclusions:**

Thus the ability of thio-ccPA 18:1 to inhibit the LPA3 receptor and ATX are key to its molecular mechanism, particularly in melanoma cells that predominantly express the LPA3 receptor. These observations necessitate further exploration and exploitation of these targets in melanoma.

## Introduction

The incidence of melanoma, the most aggressive form of skin cancer, is rising faster than any other cancer in the U.S. with a 619% increase from 1950 to 2000 [1]. While mortality from many cancers is in decline, melanoma of the skin is among only three types, including liver and esophageal, with increasing mortality among males in the U.S. [2]. Although remarkable strides in research, prevention and treatment continue to reduce cancer-related mortality overall, the mortality from melanoma is expected to rise due to the combination of increasing incidence and lack of effective therapies. Factors that increase melanoma susceptibility include accumulating genomic mutations from environmental sun exposure, a decrease in keratinocyte stem cell proliferation capacity, a decline in the regeneration ability of the skin and evolving changes in cellular signaling [3].

Advanced metastatic melanoma has an alarming average survival of only 6 to 10 months with less than 5% of patients living 5 years after diagnosis [4]. Unfortunately FDA-approved chemotherapy and immunotherapy used against advanced metastatic melanoma such as dacarbazine (DTIC), interferon (IFN) and interleukin-2 (IL-2) do not significantly improve patient outcomes in the majority (>80%) of patients [5]. Thus, more basic research is desperately needed to develop new, more effective therapeutic strategies for this disease.

The potential involvement of the lysophosphatidic acid (LPA) signaling pathway in melanoma was hypothesized when autotaxin (ATX, ENPP2) was demonstrated to be identical to a motility-stimulating factor secreted by melanoma cells [6]. ATX is the enzyme that generates the main extracellular pool of LPA [7]. LPA is a normal lipid constituent of biological fluids with a wide range of molecular signaling and resultant cellular outcomes [8,9]. LPA has been proposed to activate at least eight known G protein coupled receptors (LPA1 [10], LPA2 [11], LPA3 [12], LPA4/GPR23 [13], LPA5/GPR92/93 [14,15], GPR87 [16], P2Y5 [17], and P2Y10 (putative dual LPA and S1P receptor) [18]. LPA has also been demonstrated to activate PPARγ [19] and participates in cross communication with tyrosine kinase receptors through as yet unclear mechanisms [20,21]. The role of LPA production, LPA receptor activation and LPA receptor expression in melanoma progression, and as potential therapeutic targets, remains poorly understood.

Cyclic phosphatidic acid (1-acyl-sn-glycerol-2,3-cyclic phosphate; cPA) is a naturally-occurring compound that was originally isolated from the lipid fraction of slime mold. cPA was initially demonstrated to have strong inhibitory activity on eukaryotic DNA polymerase α, but not β or γ [22]. However, cPA exhibits multiple other actions in mammalian cells. For example, cPA prevents tumor cell migration through its ability to downregulate active RhoA and thus the downstream autophosphorylation of focal adhesion kinase [23]. Previously we demonstrated that carba analogues of cyclic phosphatidic acid (ccPA) potently inhibit ATX activity, LPA synthesis and metastatic melanoma progression in vivo [24]. Interestingly, ccPA compounds demonstrate anti-metastatic effects accompanied by inhibition of RhoA activation. This effect is not due to inhibition of LPA receptor activation [25], suggesting that inhibition of ATX and subsequent LPA production represents a critical target.

We have developed the next generation of ccPA compound, the stabilized analogue thio-ccPA 18:1, as a mechanistic probe and potential therapeutic modality. Thio-ccPA 18:1 is a phosphonothioate analogue of ccPA with an enhanced ability to inhibit ATX activity (89% at 10 μM) [26]. Thio-ccPA 18:1 is also unique due to its action as a selective inhibitor of LPA receptors, blocking LPA1 and LPA3, with no effect on LPA2 [26,27]. Thio-ccPA 18:1 has not demonstrated any detectable agonist-related activation of the LPA receptors examined, including LPA1, LPA2 or LPA3 [26].

Herein we tested the potential of thio-ccPA 18:1 as a melanoma therapeutic in vitro and as a probe of relative efficacy of inhibition of ATX, LPA1 and LPA3. We observed that thio-ccPA 18:1 reduces viability in the highly aggressive B16F10 model for metastatic disease progression. Our data demonstrates that thio-ccPA 18:1 directly inhibits the growth and viability of B16F10 melanoma cells, as well as two commonly used human melanoma cell lines, A375 and MeWo. Although ATX inhibition contributes greatly to therapeutic efficacy against melanoma [24], the effect of thio-ccPA 18:1 on viability is not only related ATX inhibition since neither LPA nor a stabilized LPA analog, R-OMPT [28] that would bypass the inhibition of ATX, were able to override the inhibitory effects of thio-ccPA 18:1. In addition, we demonstrated that inhibition of LPA3 by siRNA also results in a decrease in cell viability in melanoma cells. These studies are the first to implicate LPA3 as a critical mediator of melanoma growth and survival, and provide evidence that LPA3 mediated receptor signaling may represent an important therapeutic target in melanoma, providing an enhanced benefit of the phosphonothionate analogue.

## Methods

### Reagents and Materials

LPA (18:1, 1-oleoyl-2-hydroxy-sn-glycero-3-phosphate and 14:0, 1-myristoyl-2-Hydroxy-sn-Glycero-3-Phosphate) and (S)-phosphoric acid mono-{2-octadec-9-enoylamino-3-[4-(pyridin-2-ylmethoxy)-phenyl]-propyl} ester was purchased from Avanti Polar Lipids Inc (Alabaster, AL). D-sn-1-O-oleoyl-2-O-methylglyceryl-3-phosphothionate (R-OMPT) was purchased from Echelon Biosciences, Inc. (Salt Lake City, UT). A375 epithelial malignant melanoma, MeWo fibroblast malignant melanoma cells and OVCAR-3, A549 and MDA-MB-231 cells were acquired from ATCC (Manassas, VA) and maintained in Cellgro RPMI (Mediatech, Inc., Manassas, VA) supplemented with 5% (MeWo and A375) or 10% (OVCAR-3, A549 and MDA-MB-231) FBS (Sigma, St Louis, MO). B16-F10 murine melanoma were the kind gift of Dr. Isaiah J. Fidler at The University of Texas MD Anderson Cancer Center, Department of Cancer Biology and maintained in DMEM (Mediatech, Inc.) supplemented with 10% FBS (Sigma). The phosphonothionate ccPA 18:1 analogue (thio-ccPA 18:1) was synthesized as previously described (24). The solid lyophilized sodium salt of thio-ccPA 18:1 was reconstituted in PBS prior to use.

### Mouse xenograft model

All animal studies were conducted in compliance with the policies and regulations of the University of Texas M.D. Anderson Cancer Center Institutional Animal Care and Use Committee (IACUC). To analyze the consequence of treating metastatic melanoma tumors with thio-ccPA in vivo, thirty C57/Bl6 mice (male, 4-6 weeks old) were injected intravenously with 5 × 10^4 ^B16F10 cells into the tail vein. Three days post injection, ten mice were randomly selected for treatment with thio-ccPA 18:1. Of this group, mice were given the indicated doses of thio-ccPA by intraperitoneal injection. Thio-ccPA 18:1 injections were repeated seven days post tumor cell injection. After 21 days all surviving mice were euthanized, gross necropsy was performed and lungs were removed for further examination for the presence of metastatic lesions. Surviving mice were N = 17 for the control and N = 10 for thio-ccPA 18:1. One murine lung was processed for pathological examination and immunohistochemistry. The other lung was examined for lesions using a dissecting microscope and imaged using a Nikon Coolpix camera (Southern Microscope, Inc., Haw River, NC). Two independent observers assessed the number of nodules present on the lungs and results were averaged. Results are means ± SE of experiments. **p < 0.01 treatment groups vs. control by Tukey's test and analysis of variance.

### Cell viability

B16F10, A375 or MeWo cells were examined for viability by seeding the indicated number of cells (1 × 10^3 ^- 25 × 10^3^) in 96-well plates in quadruplicates. Cells were allowed to attach to the plate for 4-8 h in 1% FCS containing medium (or 10% FCS containing medium where indicated) prior to stimulation with PBS, 10-250 μM thio-ccPA 18:1, FBS or 0.1-10 μM 18:1 LPA where indicated. In some experiments, cells were transfected with the indicated ON-TARGETplus SMARTpool siRNA reagent (Dharmacon, Lafayette, CO) and DharmaFECT (Dharmacon) for 48 h (see below for details). CellTiter™ Blue reagent (Promega, Madison, WI) was added to plates and cells were incubated at 37°C to assess viability as previously described [29]. Images of individual wells of 96-well plates were acquired using a 12 megapixel Nikon Coolpix camera (Southern Microscope, Inc., Haw River, NC). All images show representative photos corresponding to quadruplicate conditions.

### Cell proliferation assay

A375 cells were seeded in quadruplicates in 96-well plates (2,000 cells/well) and allowed to attach for 8-16 h. Cells were then placed in 1% serum-containing medium and Transfected with the indicated indicated ON-TARGETplus SMARTpool siRNA reagent (Dharmacon)for 48 h. Proliferation was assessed as previously described using crystal violet [29]. Experimental groups were compared with siRNA negative control (Applied Biosystems, Foster City, CA) and Mock transfected controls. Results are means ± SE of experiments. *p < 0.05 treatment groups vs. control by Bonferroni's test and analysis of variance.

### Small interfering RNA (siRNA) transfection

We down regulated individual LPA receptor expression by using sequence-specific siRNA purchased from ON-TARGETplus SMARTpool siRNA reagents (Dharmacon). The cells were transfected according to the manufacturer's protocol using either reagents DharmaFECT (Dharmacon) or the X-tremeGENE siRNA transfection reagent (Roche, Palo Alto, CA). Negative control siRNA (control) was purchased from Applied Biosystems (Foster City, CA). Expression levels of gene knockdown were optimized as previously described [29,30].

### Assessment of siRNA transfection

Cells were transfected with SMARTpool siRNA reagents (Dharmacon), which contain four different siRNA, each consisting of 21 base pairs. The siRNA was extracted separately from the media and cells and analyzed by ion chromatography using UV detection. Samples of the cell medium and RNA isolated from transfected cells were collected after 0, 6, 10 and 24 h. Along with ion chromatography showing siRNA inside the cell, RNA and visual observations of cells also corroborated successful transfection targeting this receptor.

### Trypan blue exclusion

The A375 and Mewo cells were seeded in 6-well dishes at a density of 10^5 ^cells/well and allowed to attach overnight. The cells were transfected with 20 nM of the indicated siRNAs using the X-tremeGENE transfection reagent (Roche, Palo Alto, CA). After 72 h of incubation, the cells from separate wells were trypsinized, cell samples were mixed with an equal volume of a solution of 0.4% Trypan Blue dye (Sigma, St. Louis, MO) just before the counting of cells. The cells from each replicate sample were immediately transferred into both grids of a Neubauer hemocytometer and the viable (dye excluding) fraction of cells in all ten squares of both grids were counted under a microscope. Cell numbers from all squares were averaged and the total number of cells for each replicate sample was determined.

### Real-time PCR

mRNA of MeWo and A375 cells was extracted with GenElute Direct mRNA kit (Sigma Aldrich, St. Louis, MO) and reverse transcribed into cDNA using Superscript III kit (Invitrogen, Carlsbad, CA) following the manufacturer's protocol. Total human skin RNA was purchased from Agilent Technologies (Palo Alto, CA). Real-time PCR was performed using the primers for LPA1, LPA2, LPA3, LPA4 and LPA5 as previously described [31] and other primers for p2y5: forward 5'- TTGTATGGGTGCATGTTCAGC-3' and reverse 5'- GCCAATTCCGTGTTGTGAAGT -3'; p2y10: forward 5'- GTTTCCTGACGTGCATCAGTC -3' and reverse 5' - AGTCCCCACAACGATCCAGAT -3' based on algorithm generated sequences from Primer Bank http://pga.mgh.harvard.edu/primerbank/[32]. Other primers used included GPR87: forward 5'- GAGCAAGTTGTTCCAGTAGTCG -3' and reverse 5' - CTTTGAAACTAAGGTCGGCAGG -3'; ATX: forward 5'- CTCGTTCCAGTCGTGTCAGA -3' and reverse 5' - CAAGATCCGGAGATGTTGGT -3'. PCR products were visualized by loading 1 μl of product onto the Agilent DNA 1000 chip in gel-dye matrix and running the chip for 35 min in the Bioanalyzer 2100 (Agilent Technologies) following the manufacturer's protocol.

### Cell morphology

B16F10 or A375 cells were seeded in quadruplicates in 96-well plates and treated with 50 μM thio-ccPA 18:1 or transfected with the indicated siRNA for 48 h prior to the examination of cell morphology. Cells were visualized using an Axiovert 40 inverted microscope (Carl Zeiss MicroImaging Inc., Thornwood, NY) and photomicrographs were captured using a Nikon Coolpix camera (Southern Microscope, Inc.).

### Gene expression analysis

For examination of variations in biomarker expression among patient datasets, a publicly-available melanoma gene expression dataset (GSE7553, N = 87) [33] was downloaded from the NCBI Entrez Gene Expression Omnibus (GEO) DataSets website http://www.ncbi.nlm.nih.gov/sites/entrez?db=gds and analyzed as previously described [34]. Box plots using the normalized gene expression were created with GraphPad Prism (GraphPad Software, Inc., La Jolla, CA).

### Evaluation of autotaxin inhibition

In order to assess the inhibition of autotaxin, an autotaxin inhibitor screening kit (Echelon Biosciences, Inc, Salt Lake City, UT) was employed as previously described [35]. Briefly, 1 mM stock solutions were made in DMSO and then diluted with water to the appropriate experimental concentration. DMSO was spiked into each reaction mixture to equalize vehicle concentrations. This assay uses a fluorescence-quenched, lysophosphatidylcholine analogue FS-3 as the autotaxin substrate and recombinant, human autotaxin purified from Sf9 insect cells as the enzyme source. Thio-ccPA 18:1 was then pre-incubated with the enzyme at 25°C for 10 min, then FS-3 was added. The rate of fluorescence increase was measured between 5- 25 min after substrate addition. In all circumstances fluorescence increase was linear during this time window. Rates were normalized to control reactions that contained all reaction components except test compound.

### Statistical analysis

Statistical differences in experimental data was determined using analysis of variance (ANOVA) followed by either Tukey's test or Bonferroni's multiple comparison test between groups, where indicated. *p < 0.05 **p < 0.01 and ***p < 0.001 indicate the levels of significance.

## Results

**Figure 1:**
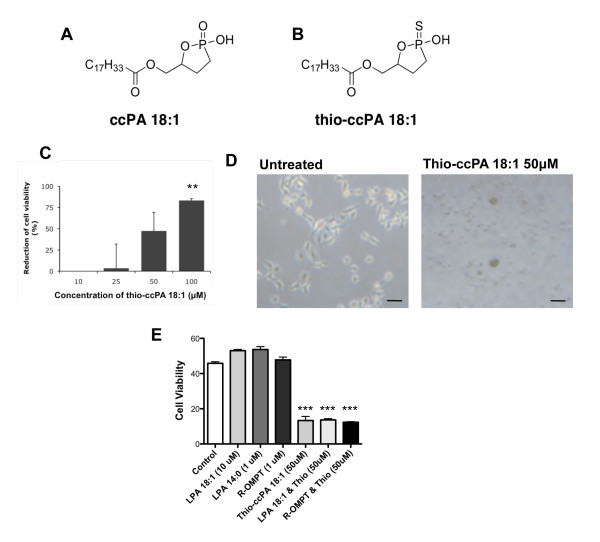
**Thio-ccPA 18:1 reduces the viability of B16F10 cells in vitro**. (A) Chemical structure of ccPA 18:1 and (B) thio-ccPA 18:1. (C) B16F10 cells were treated with increasing concentrations (10-100 μM) of thio-ccPA 18:1 and analyzed for cell viability after 48 h. The graph presents the data as the percentage of reduction in cell viability (% of PBS control). **p < 0.01 vs. control by Bonferroni's t-test and analysis of variance. (D) B16F10 cells were either untreated (control) or treated with thio-ccPA 18:1 (50 μM). Images demonstrate the difference in B16F10 cell morphology after 48 h treatments with 50 μM thio-ccPA 18:1. (E) B16F10 cells were either untreated (control) or treated with LPA 18:1 (10 μM), LPA 14:0 (1 μM), R-OMPT (1 μM), thio-ccPA 18:1 (50 μM) or a combination of these as shown. Cell viability was assessed after 48 h. ***p < 0.001 vs. control by Bonferroni's t-test and analysis of variance.

Previous observations that carba analogues of ccPA-treated animals have reduced lesions in the lungs are striking [24] and warrant further investigation. Thus, the next generation compound was synthesized based on enhanced metabolic stability of the carbacylic structure (Fig. 1A) and improved receptor binding properties. We thus hypothesized that the phosphonothionate analogue of carba cyclic phosphatidic acid, thio-ccPA 18:1 (Fig. 1B), would have interesting biological properties related to receptor binding and could be used to explore approaches against melanoma progression.

In order to assess the most fundamental question, whether thio-ccPA 18:1 had an effect on melanoma cell viability, we examined the viability of metastatic melanoma B16F10 cells in vitro in the presence or absence of thio-ccPA 18:1. No significant reduction in viability was observed at 10 or 25 μM thio-ccPA 18:1 after 48 h; however, concentrations of 50 μM and above induced a dramatic reduction in viability, approximately 49% at 50 μM and 85% at 100 μM (**P = 0.01 vs. PBS control Fig. 1C). Thio-ccPA 18:1 (50 μM) treated B16F10 appeared small and rounded compared with untreated controls that were flattened and exhibited lamellipodia protrusions (Fig. 1D). Visual examination of individual wells treated with 100 μM detected few attached cells after 48 h (data not shown).

Thio-ccPA 18:1 targets several components of the LPA signaling pathway. It is an effective inhibitor of ATX, similar to other cyclic phosphatidic acid analogues, but it also is a direct antagonist of the LPA1 and LPA3 receptors [27]. If ATX activity is the only important target of thio-ccPA 18:1 [6], then exogenous LPA should override the effects of thio-ccPA 18:1 [24]. In order to test this hypothesis, we pre-treated the melanoma cells with LPA 18:1 (10 μM) or the metabolically stabilized LPA analogue D-sn-1-O-oleoyl-2-O-methylglyceryl-3-phosphothionate (R-OMPT) (1 μM) [28] prior to treatment with thio-ccPA 18:1 (50 μM). Pre-treatment of LPA or R-OMPT was unable to bypass the reduction in cell viability induced by thio-ccPA 18:1 (Fig. 1E). This suggests that additional targets, complementary to ATX inhibition, contribute to the ability of thio-ccPA 18:1 to reduce melanoma cell viability.

We next tested whether thio-ccPA 18:1 inhibits the viability of human melanoma cells. This was done in order to better represent translational applications of the phosphonothionate analogue to humans, to broadly examine multiple melanoma cell lines and their responses to thio-ccPA 18:1 and because B16F10 cells are hyper-sensitive to fluctuations in the concentration of serum contained in medium which could reflect an oncogenic addiction to growth factors or bias in our in vitro data [3] (Fig. S1A, Additional file 1). Neither the A375 or MeWo cells are sensitive to serum starvation (Fig. S1B, Additional file 1), in contrast with B16F10 cells (Fig. S1B and C, Additional file 1).

**Figure 2:**
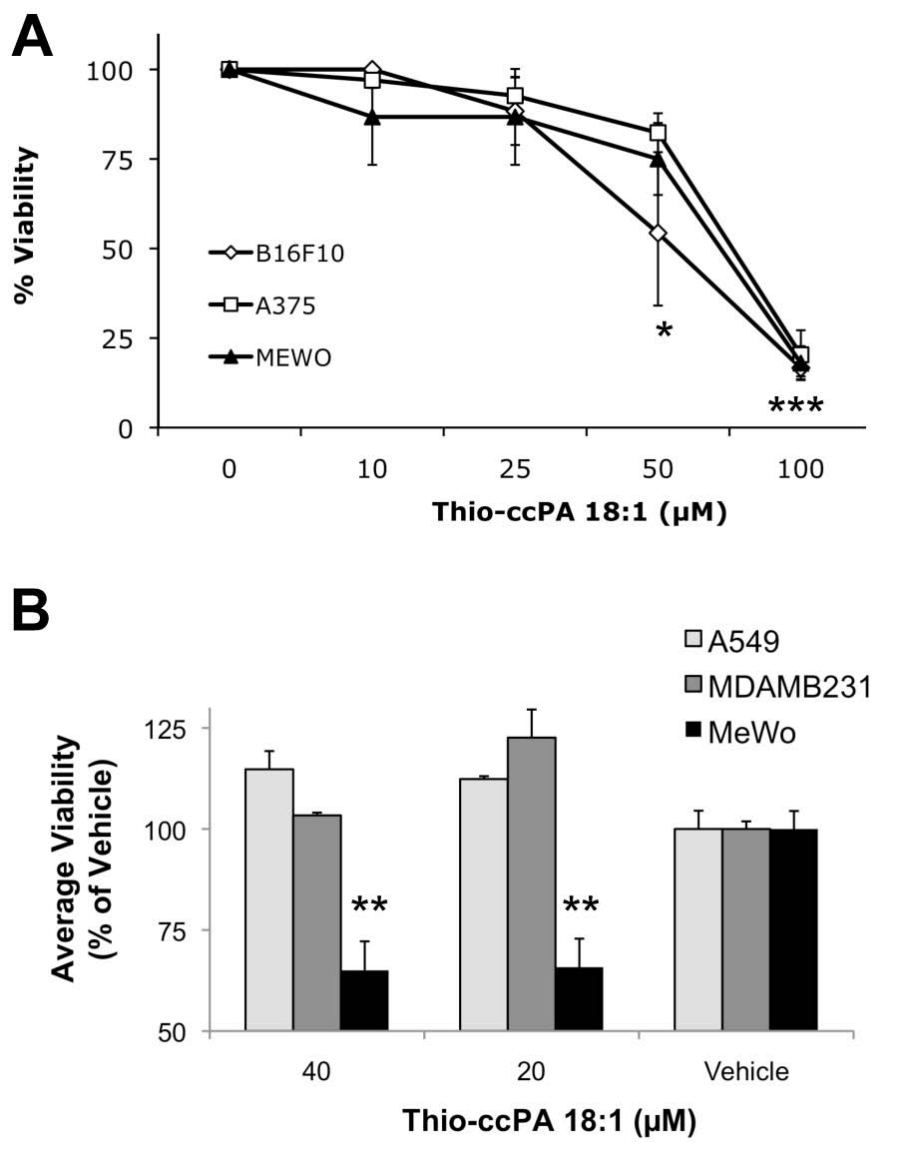
**Cell line comparison of viability reduction by thio-ccPA 18:1**. (A) B16F10, A375 and MeWo melanoma cells were treated with increasing concentrations (0-100 μM) of thio-ccPA 18:1 for 48 h and examined for viability. *p < 0.05 and ***p < 0.001 vs. control by Bonferroni's t-test and analysis of variance. (B) A549, MDA-MB-231 and MeWo melanoma cells were treated with either 40 μM or 20 μM of thio-ccPA 18:1 and examined for viability. Results show the percent of viability compared to vehicle (dH_2_O). **p < 0.01 vs. vehicle by Bonferroni's t-test and analysis of variance.

Although the three melanoma cell lines represent distinct and common genetic abnormalities observed in melanoma, B16F10 (RAS), A375 (activating B-RAF, constitutively active MAPK) and MeWo (no BRAF or NRAS), they exhibited similar decreases in cell viability in the presence of increasing concentrations of thio-ccPA 18:1 (Fig. 2A). This suggests the existence of a "common" mechanism exploited by thio-ccPA 18:1 on the LPA signaling pathway in these melanoma cells. Furthermore, this mechanism is not shared by all tumor cell types since A549, a lung cancer cell line, and MDA-MB-231, a breast cancer cell line, are insensitive to the effects of thio-ccPA 18:1 (Fig. 2B). A549 cells express LPA1 > LPA4 > LPA2 receptors and the MDA-MB-231 cells express LPA1 >> LPA2 receptors. Both cell lines express low levels of ATX and no LPA3 receptors [36].

In addition to its inhibition of ATX (Fig. S2, Additional file 2), thio-ccPA 18:1 is an LPA1/3 receptor antagonist [27]. We wanted to determine the importance of the receptor antagonism to the efficacy of the analogue, especially considering that the drug is insensitive in cells that lack the LPA3 receptor. We therefore assessed whether sequentially inhibition of individual LPA receptors affected melanoma cell viability. Specific inhibitors targeting all LPA receptors individually do not exist [37]; thus, we used siRNA to target individual LPA receptors. With siRNA we can consistently reduce the amount of LPA receptor expression approximately 60% or greater [29] (Fig. S3A, Additional file 3) without activating compensatory receptor expression mechanisms [30]. We have previously established that LPA receptor knock-down reduces LPA-mediated functions of LPA receptors in vitro and in vivo [29,30]. Here we also assessed the ability of siRNA to enter the cell from the transfected medium using ion chromatography using UV detection. Strikingly, we detected siRNA inside MeWo cells 6 h after transfection and this was detected in subsequent time points of 10 h and 24 h (Fig. S3B Additional file 3).

**Figure 3:**
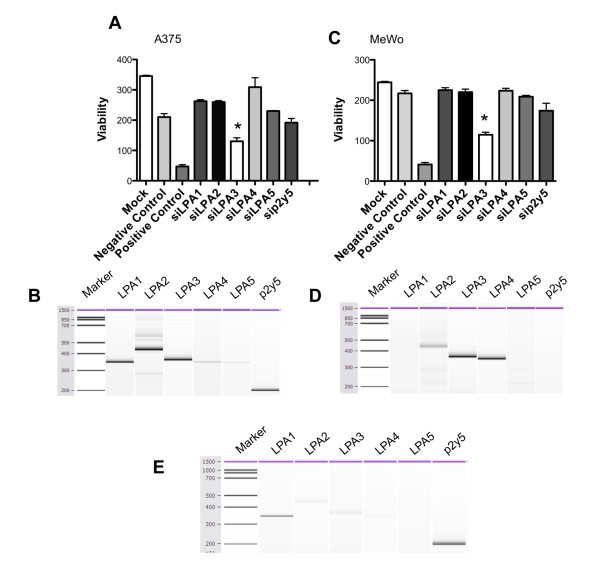
**The LPA3 receptor mediates viability in A375 and MeWo melanoma cells**. Individual LPA receptors were targeted with siRNA to sequentially inhibit receptor expression. (A) A375 cells were transfected for 48 h with the indicated siRNA and examined for viability. *P < 0.05, vs. Mock in A375 by Bonferroni's t-test and analysis of variance. (B) RT-PCR showing LPA receptor expression in A375 cells visualized using the Bioanalyzer 2100. (C) MeWo cells were transfected for 48 h with the indicated siRNA and examined for viability. *P < 0.05, vs. Negative Control (RISC-free) in MeWo. (D) RT-PCR showing LPA receptor expression in MeWo cells visualized using the Bioanalyzer 2100. (E) RT-PCR showing LPA receptor expression in normal human skin visualized using the Bioanalyzer 2100.

We next transfected A375 cells with siRNA for the LPA receptors and detected a significant reduction in cell viability when the LPA3 receptor expression was reduced (Fig. 3A). The expression of verified LPA receptors in A375 cells is LPA1, LPA2, LPA3, p2y5 >> LPA4, LPA5 (Fig. 3B), demonstrating that the LPA3 receptor is present in A375 cells. Similar results were achieved using siRNA in MeWo cells (Fig. 3C). The expression pattern of LPA receptors was very different in MeWo cells, LPA3, LPA4 >> LPA2 (Fig. 3D) compared to A375 cells. The commonalities between the two cell lines are expression of the LPA2, LPA3 and LPA4 receptors. This pattern is not commonly observed among cancer cell lines [36]. This also led us to examine LPA receptor expression in normal skin where we detected expression of p2y5 and LPA1 receptors and little (LPA2, LPA3) to no (LPA4, LPA5) expression of other receptors (Fig. 3E).

**Figure 4:**
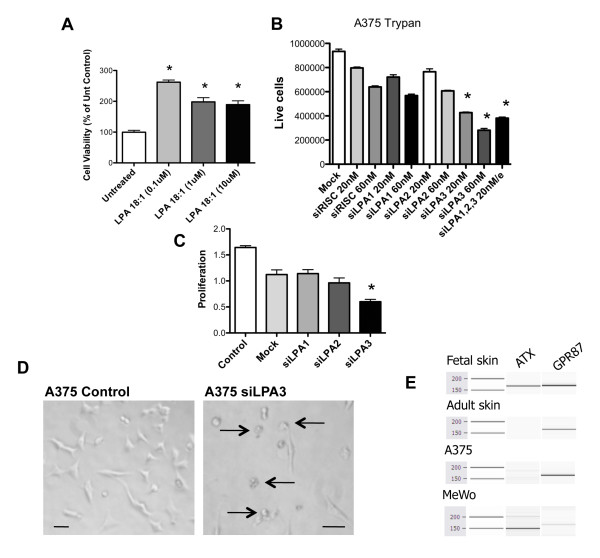
**Knockdown of the LPA3 receptor induces cell death in A375 melanoma cells**. (A) LPA 18:1 treatment (0.1-10 μM) of A375 cells in serum free medium for 48 h enhances viability. *p < 0.001 vs. untreated control. (B) A375 cells were transfected for 48 h with the indicated siRNAs and assessed for membrane integrity and cell death using trypan blue exclusion assay. Cell numbers reflect the cells with intact membranes. *P < 0.001, comparing siRISC 60 nM vs. treatment conditions. (C) Transfection of siRNA targeting the LPA receptors in A375 cells for 48 h demonstrates that reducing the expression of the LPA3 receptor decreases the number of live cells assessed by crystal violet staining. *p < 0.05 vs. control. (D) Photomicrograph images demonstrating changes in cell morphology after 48 h of siLPA3 transfection. (E) RT-PCR demonstrating the level of expression of ATX and GPR87 in fetal skin, normal human skin, A375 and MeWo cells.

To further investigate the effects of thio-ccPA 18:1 on melanoma cells and determine whether targeting the LPA3 receptor has a complementary role to ATX inhibition, we examined LPA receptor-mediated cell viability using A375 melanoma cells. Cells were treated for 48 h in serum-free medium with LPA 18:1 (0.1 - 10 μM) and assessed for cell viability. Indeed, the addition of LPA alone enhanced the overall number of A375 cells (Fig. 4A). We next assessed cell death by measuring the integrity of the cell membrane using trypan blue exclusion and A375 cells transfected with increasing concentrations and combinations of siRNA for the LPA1, LPA2 or LPA3 receptors. Only cells transfected with siLPA3 (20 nM, 60 nM or combination) produced conditions that significantly affected the number of live cells (Fig. 4B). A375 cells transfected with 20 nM or 60 nM siLPA3 contained 45% and 30%, respectively, of live cells compared with Mock control. Cell number was further assessed using crystal violet (proliferation) staining and (20 nM) siLPA3. We measured a decrease of 70% and 50% in the number of cells transfected with siLPA3 compared with untreated (Control) and Mock control, respectively (Fig. 4C). A375 cells transfected with siLPA3 have rounded cell morphology that is distinct from normal control cells (Fig. 4D) but reminiscent of thio-ccPA 18:1 treated cells shown in (Fig. 1E). To assess whether ATX was a major contributor to viability in A375 cells, we measured the amount of expression using RT-PCR. We were barely able to distinguish a marginal level of ATX in A375 cells, although fetal skin and MeWo cells did express detectable levels of ATX (Fig. 4E). Taken together, this suggests that inhibition of the LPA3 receptor signaling reduces the viability of A375 melanoma cells and may be complimentary to ATX inhibition as a dualistic mechanism of action of thio-ccPA 18:1.

**Figure 5:**
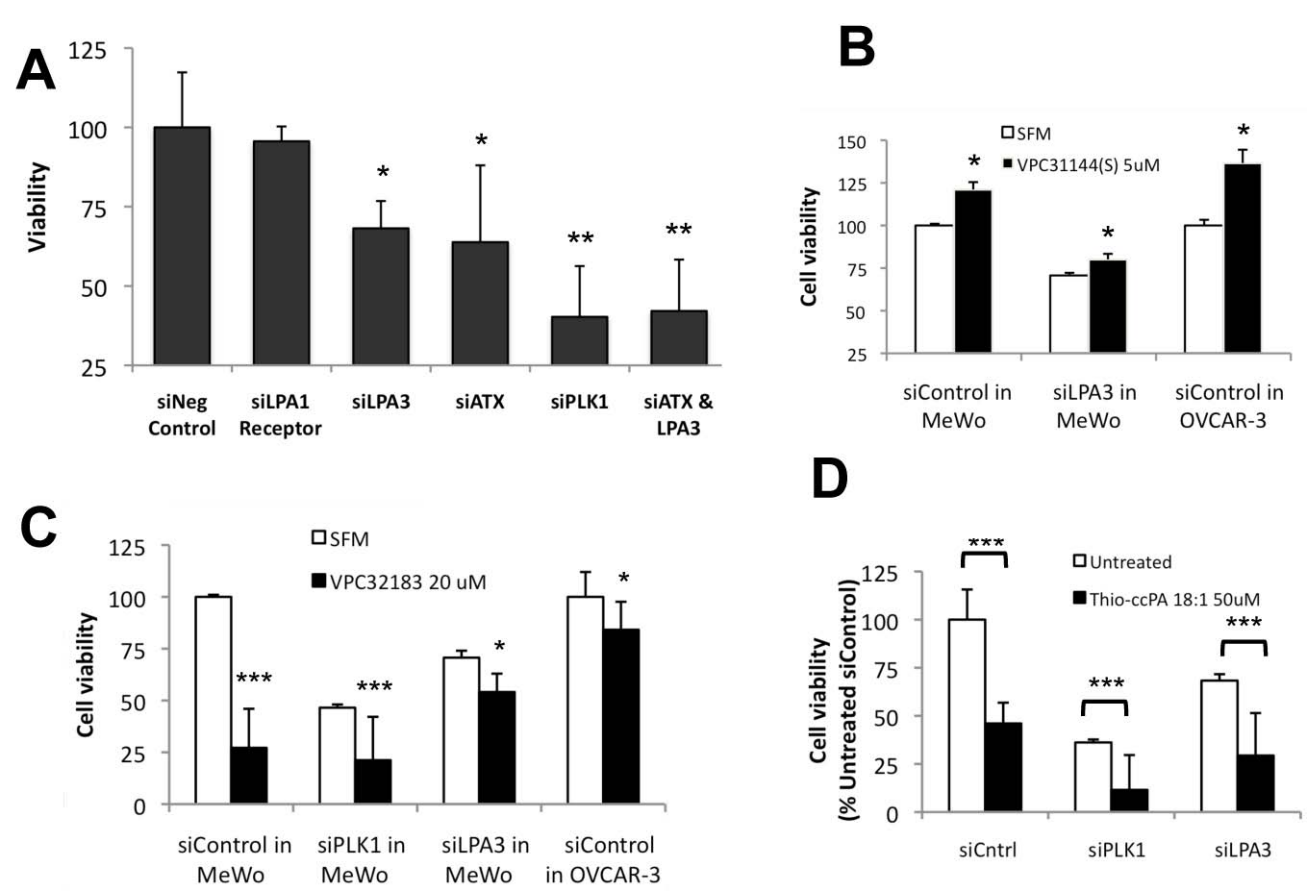
**Inhibition of the LPA3 receptor using siRNA knockdown or LPA3 antagonists reduces viability of MeWo melanoma cells**. (A) MeWo cells were plated in 96-wells and transfected with the indicated siRNAs for 24 h prior to the assessment of viability. The control, siNegative (non-targeting siRNA), was normalized to 100%. *p < 0.05 and **p < 0.01 vs. siNegative control. (B) The agonist, VPC31144(S) 5 μM was added to either MeWo cells or OVCAR-3 cells in 96-well plates in serum-free medium and compared to control, serum-free medium alone. *p < 0.05 comparing agonist to untreated in each group. (C) MeWo or OVCAR-3 cells were plated in 96-wells prior to transfection with the indicated siRNA conditions. The LPA1/3 antagonist, VPC32183 (20 μM) was added to the cells and viability was assessed. *p < 0.05, ***p < 0.001 comparing treated vs. untreated in each transfection condition. (D) MeWo cells were plated in 96-wells prior to transfection with the indicated siRNAs for 24 h and treatment with Thio-ccPA 18:1 (50 μM) in serum-containing medium. Cell viability was assessed and the siNegative control was normalized to 100%. ***p < 0.001 comparing treated vs. untreated in each transfection condition.

The expression pattern observed in MeWo cells (ATX and the LPA3 receptor) makes this the quintessential line for confirming the efficacy of thio-ccPA 18:1. Inhibiting the abundance of these two transcripts and then assessing cell viability demonstrated that either siLPA3 or siATX was capable of reducing viability (Fig. 5A). Combining siLPA3 and siATX further reduced viability over each individually and was comparable to siPLK1, a positive control for siRNA transfection that results in termination of cells.

We next asked whether the LPA3 receptor plays a large role in mediating LPA-induced cell viability in MeWo cells. A selective LPA receptor agonist induced an enhancement in viability by 21% under control conditions in MeWo cells, 37% under control conditions in OVCAR-3 cells but only 9% after MeWo cells were transiently transfected with siRNA against the LPA3 receptor and after overnight treatment with the agonist (Fig. 5B). We further inhibited the LPA3 receptor using a selective antagonist for LPA1/3, VPC32183, in MeWo cells (which do not express the LPA1 receptor). We treated the MeWo cells for 48 h and measured approximately 75% decrease in cell viability (Fig. 5C). The marked reduction in viability was blunted after transiently transfecting MeWo cells with siRNA against the LPA3 receptor or by using the OVCAR-3 cell line, which expresses multiple LPA receptors. This suggests that LPA3 expression, but likely lack of LPA2 expression, is required for response to the antagonist VPC32183. The effect on blunting the cellular response to the compound was not observed after transiently transfecting MeWo cells with siRNA against the LPA3 receptor and treating with thio-ccPA 18:1 (Fig. 5D). In the presence of LPA3 receptor knock-down, thio-ccPA 18:1 further reduced cell viability, suggesting multiple targets induce the effects of thio-ccPA 18:1. We also noted the response of VPC32183 was reduced by the presence of serum (Fig. 5E). Our data suggests the unique pattern of expression in MeWo cells (ATX and the LPA3 receptor, without the LPA1 or LPA2 receptors) provides a quintessential model for achieving a response to thio-ccPA 18:1 and represents a type of tumor that is susceptible to the actions of thio-ccPA 18:1.

**Figure 6:**
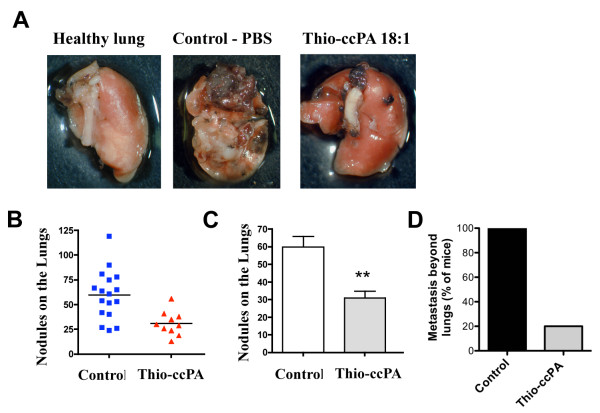
**Thio-ccPA 18:1 reduces metastatic melanoma lesions in murine lungs**. C57/BL6 mice (N = 27) were injected with 5 × 10^4 ^B16F10 cells into the tail vein. Thio-ccPA 18:1 was administered on days 3 and 7 during the 21-day study. (A) Necropsy images of left lung lobes demonstrate the presence of tumor. Quantification of tumor revealed a reduction in nodules on the lungs shown as both scatter (B) and bar (C) graphs. **p < 0.01 comparing control (PBS) with thio-ccPA 18:1 treated groups. (D) Quantification of additional lesions detected on organs outside the lungs (kidney, liver, pancreas and intestines) and presented as a percentage of mice in each group (Control N = 17, 100%; thio-ccPA 18:1 N = 2 of 10, 20%; thio-ccPA 18:1).

In order to determine whether thio-ccPA 18:1 would influence metastatic melanoma tumors in vivo, we tested its efficacy using the B16F10 metastatic melanoma mouse model [38]. We injected B16F10 metastatic melanoma cells into the tail vein of C57/Bl6 mice. Animals were intraperitoneally injected on days 3 and 7 after intravenous cell injection with 200 μg (10 mg/kg per dose) concentrations of thio-ccPA 18:1 or PBS (Control). Mice were then sacrificed 21 days after the initial injection of B16F10 cells, and tissues were fixed and analyzed for the number of metastases. Treatment with thio-ccPA 18:1 significantly reduced the number of pulmonary metastases in mice as compared to the control treatment (p < 0.01, Fig. 6A, B and 6C). In addition to the effect on pulmonary metastasis, all control animals (N = 17) had metastatic lesions to organs outside of the lungs, whereas only twenty percent (N = 2/10) of thio-ccPA 18:1 treated animals had detectable non-pulmonary metastases (Fig. 6D).

**Figure 7:**
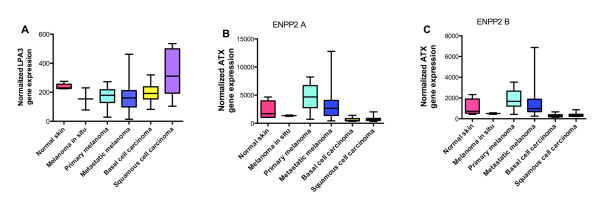
**A subset of melanomas express high levels of the LPA3 receptor and ATX**. Gene expression microarray data was downloaded from the NCBI Gene Expression Omnibus containing patient specimen datasets (GSE7553, N = 87) [33]. The genes (A) LPA3 and (B and C) ATX (ENPP2 A and B) were selected and the data was converted into box plot graphs to demonstrate the range of expression levels among these genes.

If expression patterns reflect biomarker signatures that confer susceptibility to thio-ccPA 18:1, we were curious how prevalent high levels of ATX and the LPA3 receptor expression are in melanoma. For this we profiled gene expression microarray data downloaded from the NCBI Gene Expression Omnibus. Among patient specimen datasets (GSE7553, N = 87) [33], gene expression analyses reveals significant variation of LPA3 receptor expression in metastatic melanoma (Fig. 7A), suggesting that not all types of advanced melanoma might be strongly susceptible to thio-ccPA 18:1. This is consistent with the variation in LPA3 receptor expression we observed among melanoma cell lines. We also detected a significantly increased level of ATX among metastatic melanoma specimens (N = 40) compared with basal cell carcinoma (N = 15), normal skin (N = 5) and squamous cell carcinoma of the skin (N = 11) (Fig. 7B and 7C). ATX expression in primary melanoma (N = 14) is also increased in comparison to melanoma in situ, basal and squamous cell carcinoma. Taken together, the data suggests that a portion of metastatic melanomas, estimated at approximately 20%, express high levels of ATX and/or the LPA3 receptor and this population represents the most appealing pool for therapeutic intervention.

## Discussion

Thio-ccPA 18:1 is a unique compound with multiple targets. Biological testing of thio-ccPA 18:1 demonstrated it is an antagonist of the LPA1 and LPA3 receptors along with its activity as an effective inhibitor of ATX [27]. Thus, the compound is described as having a "one-two-punch" [39] because it inhibits the generation of LPA and the initiation of LPA-mediated signaling through LPA1 and LPA3 receptors.

In this study, we demonstrate the in vitro and in vivo efficacy of thio-ccPA 18:1 and describe its dualistic mechanism of action, responsible for decreasing in vitro viability in melanoma cells. Either addition of thio-ccPA 18:1 or siRNA for the LPA3 receptor significantly reduces A375, MeWo and B16F10 melanoma cell viability in vitro suggesting that the effects may be generalizable to melanoma cells. In addition, siLPA3 reduces the membrane integrity and proliferation of A375 cells and alters cell morphology. Neither thio-ccPA 18:1 nor siLPA3 induced nuclear fragmentation (unpublished observations), suggestive of a non-apoptotic mechanism of reduced cell viability. We also show that in vivo treatment with thio-ccPA 18:1 inhibits B16F10 cell metastatic lesions that develop in the lungs and prevents the spread of metastases to distant organ.

Previous studies have examined carba analogues of cyclic phosphatidic acid that inhibit ATX activity [24,25]. Our collaborative study suggested that ATX is a major mediator of melanoma metastasis in vivo and cancer cell invasion in vitro and that these analogues work effectively by inhibiting ATX activity, without receptor antagonism [24]. Indeed, studies demonstrating the anti-metastatic capability of ccPA compounds suggested this effect did not require inhibition of LPA receptor activation [25]. However, these observations did not address whether coupling ATX inhibition with receptor blockade would be more effective than targeting ATX alone or whether different populations of melanoma cells have specific LPA receptor targeting susceptibilities.

Other studies have confirmed that ATX inhibitors reduce melanoma cell migration and invasion [40]. ATX continues to be an important therapeutic target for cancer because it may be involved in protecting cells from apoptosis-induced chemotherapy [41]. Besides melanoma, ATX may also play a role in the motility of glioblastoma cells, which may be critical due to the high expression of ATX among the CNS and glioblastoma multiforme [36]. We have also shown that carba analogues of cyclic phosphatidic acid inhibit the LPA-stimulated motility of prostate cancer cells [29]. In other diseases, ATX may provide a useful serum biomarker for follicular lymphoma [42] and chronic hepatitis C [43].

Unlike ATX, there are few studies devoted to the investigation of the efficacy of targeting the LPA3 receptor. Our current study enhances the biological understanding of LPA3 receptor function and these findings are a major novelty of this study. Our previous study suggested the increased presence of any of the LPA1, LPA2 or LPA3 receptors enhances tumorgenicity and aggressiveness of ovarian cancer [30]. Thus far, the only known independent function of the LPA3 receptor occurs in reproductive biology where it regulates embryo implantation and spacing [44]. Another study suggests women with endometriosis have decreased expression of the LPA3 receptor in the endometrium, suggesting a hypothesis for their observed subfertility [45].

The study presented herein is the first to suggest that the LPA3 receptor plays a crucial role in melanoma cell viability in vitro. This is the first study to characterize this unappreciated mechanism of action of the novel compound, thio-ccPA 18:1. It fills a gap in our knowledge about novel ccPA compounds designed to inhibit LPA signaling because it highlights a role for receptor antagonism, in addition to blocking ATX activity. The fact that compounds which inhibit ATX are potent agents against tumor progression is intriguing but leads to an obvious mechanistic question - why does inhibiting the production of LPA have potent biological effects? Based on our data, we hypothesize that the lack of LPA production resultant from ATX inhibition leads to a critical reduction of LPA receptor-mediated survival signaling required for viability among specific populations of melanoma cells.

One limitation of our study surrounds the intrinsic properties of siRNA and their utility. We used gene silencing to target individual LPA receptors to verify the receptor antagonist properties of thio-ccPA 18:1; however, off-target activity of siRNAs can lead to complex interpretations of observed phenotypes. Studies using microarray gene expression profiling previously supported the notion that induction of siRNA would specifically silence the intended target but it is now acknowledged that off-target activity can occur and is not ameliorated by decreasing the siRNA concentration [46]. In our study we cannot rule out the possibility that siRNA of the LPA3 receptor (or siRNA for ATX or any other LPA receptor) may have off-target effects through microRNA-like down regulation; however, we are using pooled siRNA reagents which reduces the overall number of off-targets through competition among siRNAs. In addition, we observe a similar reduction in cell viability using either thio-ccPA 18:1 or VPC32183. This suggests both the receptor antagonism of the compounds and siRNA are all targeting the same receptor and the phenotype is identical. Therefore, this limitation is not a major concern since specific antagonists targeting the LPA3 receptor significantly reduced cell viability in MeWo cells. Finally, we used a novel approach of ion chromatography and UV detection to demonstrate that the siRNA was incorporated into the cells. This technique showed that each of the four siRNAs contained in the SMARTpool entered the cells.

The findings of our study have several future therapeutic and translational potentials. The data suggests targeting the LPA signaling pathway has efficacy against tumor progression, in particular against metastatic melanoma. It compliments previous studies [24,25] and strengthens a need for further research using melanoma models that we are currently undertaking. The pattern of LPA receptor expression in melanoma cells may be important to understanding how the elimination of one receptor, which is presumably part of a redundant signaling family, results in a marked decrease in viability. For example, Lee et al. suggested that it is not merely the expression of the LPA1 receptor which controls LPA-mediated cell motility as previously suggested [47], but the lack of LPA4 receptor expression that affects motility as it would otherwise regulate function of the LPA1 receptor [48]. A similar dualistic mechanism could account for LPA-mediated cell viability and that expression of this yet unknown counter-regulating protein or receptor is absent in these melanoma cells. Finally, it is important to clarify the molecular mechanism of action of pharmaceutical compounds to improve lead compound design and predict potential side effects that may appear during clinical and preclinical trials so they can be monitored and managed appropriately.

## Competing interests

GP is a scientific advisor for Echelon Biosciences, Inc. and RxBio.

DM is at Echelon Biosciences, Inc.

## Authors' contributions

MKA, VG, WJ and MMM carried out the in vitro molecular biology testing and data analysis. GJ, YX and GDP carried out the design and synthesis of the compounds. DM measured the autotaxin activity. ACM and MGB carried out the siRNA analysis by ion chromatography using UV detection. MMM, SX and HH carried out the animal studies. MMM carried out the bioinformatic studies. MMM and MAD conceived the study, and participated in its design and coordination. MMM wrote the paper with GBM, MAD and GDP contributing improved intellectual concepts for manuscript revisions.

## Supplementary Material

Additional file 1 **Figure S1. B16F10 cells are highly sensitive to serum conditions**. (A) B16F10 cells were grown in medium with increasing concentrations of fetal bovine serum (0-20%) and assayed for cell viability after 24 h. Results are presented as the average percent, normalized to 0% serum conditions. ***p < 0.001 vs. 0% serum by Bonferroni's t-test and analysis of variance. (B) B16F10, A375 and MeWo cells were grown overnight in 10% serum-containing medium (SCM) or serum-free medium (SFM). Cells were counted in each condition and results are presented as the average cell number. ***p < 0.001 SCM vs. SFM by Bonferroni's t-test and analysis of variance. (C) Image represents the difference in the number of B16F10 cells grown overnight in SCM or SFM. Click here for file

Additional file 2 **Figure S2. Inhibition of the lysophospolipase D activity of human recombinant autotaxin by the thio-ccPA 18:1 analogue**. Thio-ccPA 18:1 was pre-incubated with the autotaxin enzyme at 25°C for 10 min, after which FS-3, a fluorescence-quenched, lysophosphatidylcholine analogue that acts as the autotaxin substrate, was added to the reaction. The rate of fluorescence increase was measured between 5-25 min after the substrate addition. Rates were normalized to control reactions that contained all reaction components except the test compound. Click here for file

Additional file 3 **Figure S3. Analysis of siLPA3 transfection in MeWo cells**. (A) MeWo cells were grown in 96-well plates overnight prior to siLPA3 transfection for 24 h. Cells were then lysed directly in 96-wells using TriReagent and RNA was isolated. Q-RT-PCR assessed the expression of MeWo control RNA in comparison to MeWo cells transfected with siLPA3. (B) MeWo cells were transfected with siLPA3 SMARTpool siRNA, which contains 4 different siRNA, each consisting of 21 base pairs. The RNA was extracted from the cells after washing in PBS at 0, 6, 10 and 24 h and analyzed by ion chromatography using UV detection. The results show 4 siRNA peaks that indicate siRNA is inside the cell. Click here for file
