# Supplementary figures and images for: Targeting melanoma growth and viability reveals dualistic functionality of the phosphonothionate analogue of carba cyclic phosphatidic acid
Source: Mol Cancer. 2010 Jun 9;9:140. doi: 10.1186/1476-4598-9-140 (PMC2895597; doi:10.1186/1476-4598-9-140)

## Slide 1
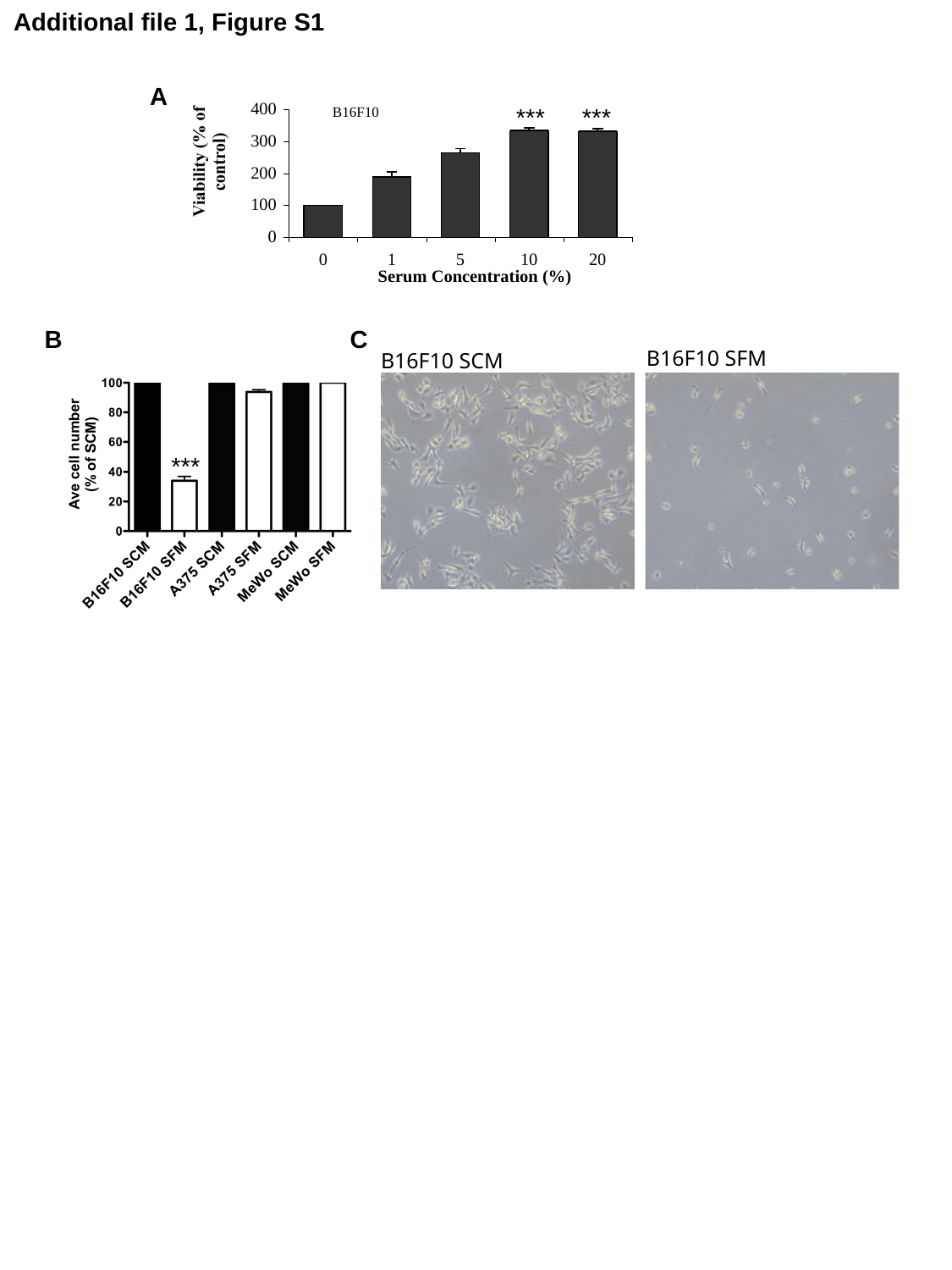

Additional file 1, Figure S1
A
***
***
B
C
B16F10 SFM
B16F10 SCM
***

Supplement: Additional file 1 — Figure S1. B16F10 cells are highly sensitive to serum conditions. (A) B16F10 cells were grown in medium with increasing concentrations of fetal bovine serum (0-20%) and assayed for cell viability after 24 h. Results are presented as the average percent, normalized to 0% serum conditions. ***p < 0.001 vs. 0% serum by Bonferroni's t-test and analysis of variance. (B) B16F10, A375 and MeWo cells were grown overnight in 10% serum-containing medium (SCM) or serum-free medium (SFM). Cells were counted in each condition and results are presented as the average cell number. ***p < 0.001 SCM vs. SFM by Bonferroni's t-test and analysis of variance. (C) Image represents the difference in the number of B16F10 cells grown overnight in SCM or SFM. [file 1476-4598-9-140-S1.PPT]

## Slide 1
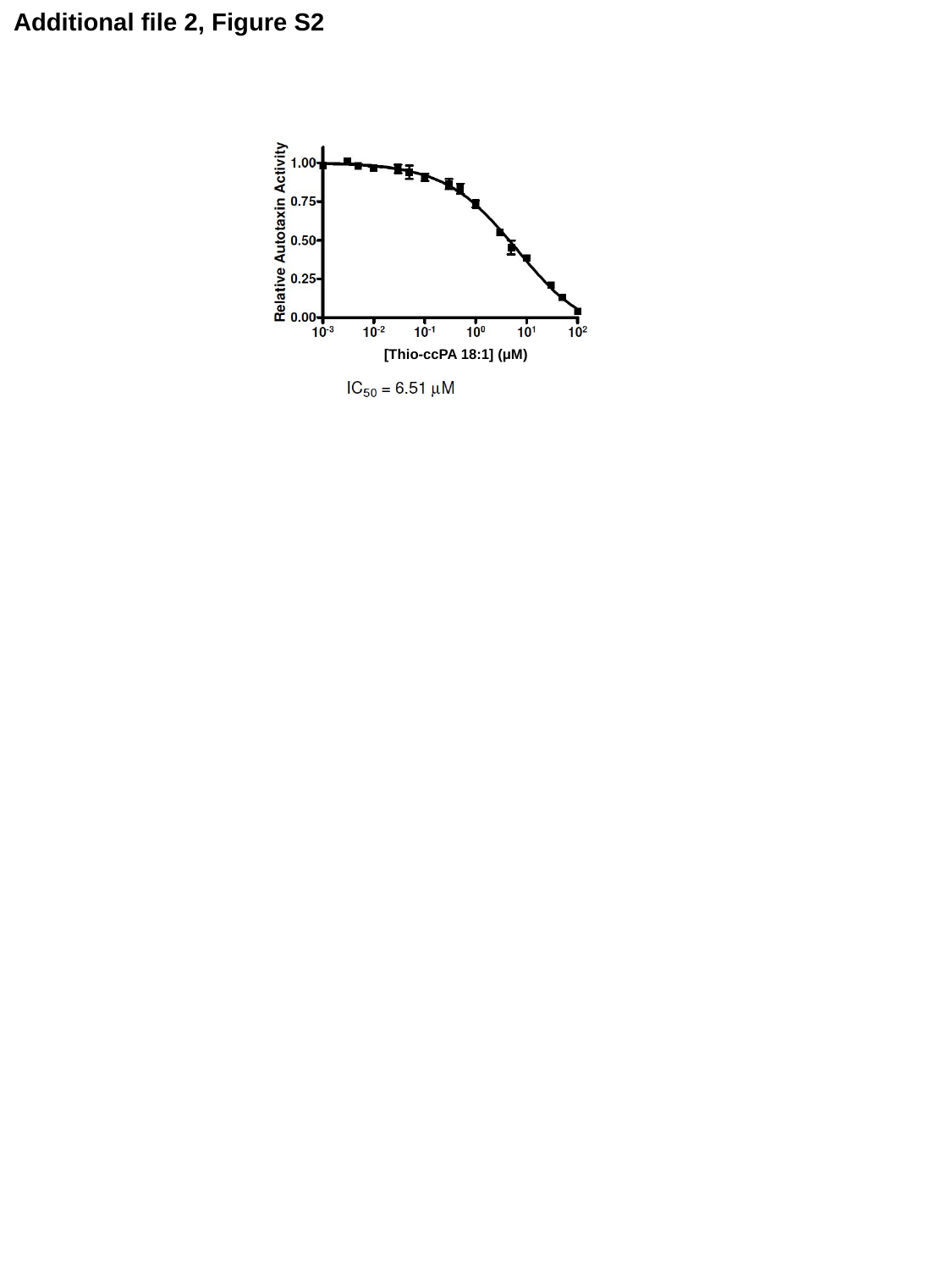

Additional file 2, Figure S2
[Thio-ccPA 18:1] (μM)

Supplement: Additional file 2 — Figure S2. Inhibition of the lysophospolipase D activity of human recombinant autotaxin by the thio-ccPA 18:1 analogue. Thio-ccPA 18:1 was pre-incubated with the autotaxin enzyme at 25°C for 10 min, after which FS-3, a fluorescence-quenched, lysophosphatidylcholine analogue that acts as the autotaxin substrate, was added to the reaction. The rate of fluorescence increase was measured between 5-25 min after the substrate addition. Rates were normalized to control reactions that contained all reaction components except the test compound. [file 1476-4598-9-140-S2.PPT]
