# Supplementary material for: Targeting melanoma growth and viability reveals dualistic functionality of the phosphonothionate analogue of carba cyclic phosphatidic acid
Source: Mol Cancer. 2010 Jun 9;9:140. doi: 10.1186/1476-4598-9-140 (PMC2895597; doi:10.1186/1476-4598-9-140)
Supplement: Additional file 3 — Figure S3. Analysis of siLPA3 transfection in MeWo cells. (A) MeWo cells were grown in 96-well plates overnight prior to siLPA3 transfection for 24 h. Cells were then lysed directly in 96-wells using TriReagent and RNA was isolated. Q-RT-PCR assessed the expression of MeWo control RNA in comparison to MeWo cells transfected with siLPA3. (B) MeWo cells were transfected with siLPA3 SMARTpool siRNA, which contains 4 different siRNA, each consisting of 21 base pairs. The RNA was extracted from the cells after washing in PBS at 0, 6, 10 and 24 h and analyzed by ion chromatography using UV detection. The results show 4 siRNA peaks that indicate siRNA is inside the cell. [file 1476-4598-9-140-S3.PPT]

## Slide 1
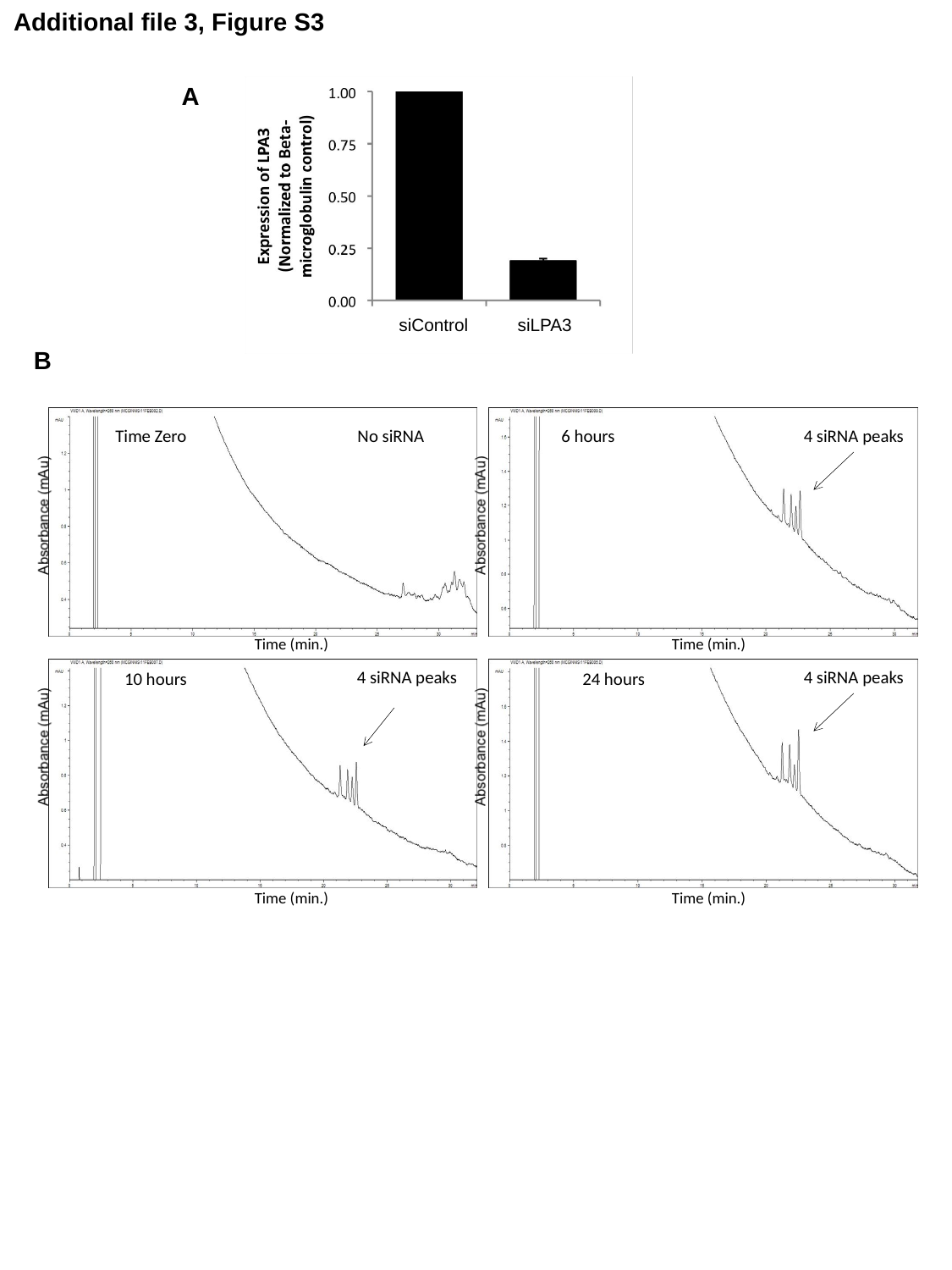

Additional file 3, Figure S3
A
siControl
siLPA3
B
Figure 1: Analysis of Edg7 siRNA extracted from SKOV3 cells at four time points
Time Zero
No siRNA
6 hours
4 siRNA peaks
Time (min.)
Time (min.)
4 siRNA peaks
4 siRNA peaks
10 hours
24 hours
Time (min.)
Time (min.)
